# Supplementary material for: Histone modifications regulate pioneer transcription factor cooperativity
Source: Nature. 2023 May 24;619(7969):378–84. doi: 10.1038/s41586-023-06112-6 (PMC10338341; doi:10.1038/s41586-023-06112-6)
Supplement: Supplementary file 1 — This file contains Supplementary Fig. 1 - the raw gels, Supplementary Tables 1 and 2 which list oligonucleotides and plasmids used in the study and Supplementary Table 3 - quantification of data. [file 41586_2023_6112_MOESM1_ESM.pdf]

nature portfolio

https://doi.org/10.1038/s41586-023-06112-6

Supplementary information

Histone modifications regulate pioneer transcription factor cooperativity

In the format provided by the authors and unedited

Supplementary Information

Supplementary Figure 1

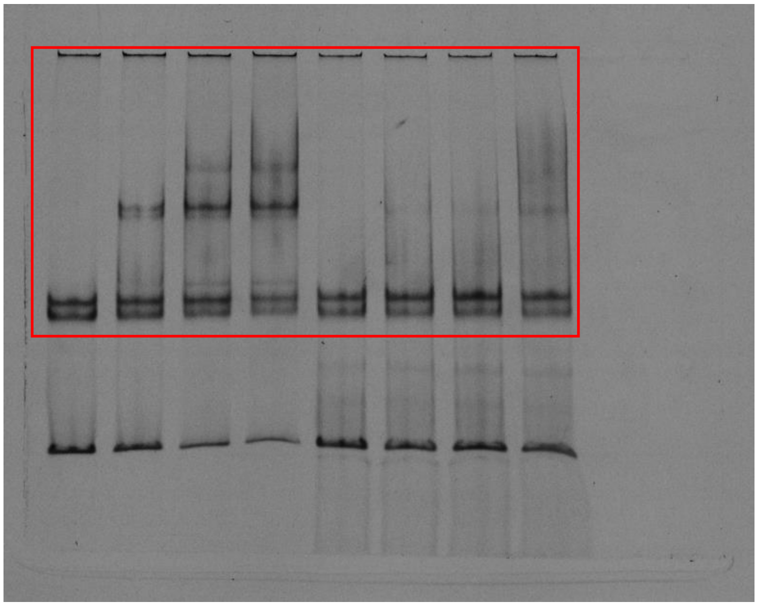

Figure 3b

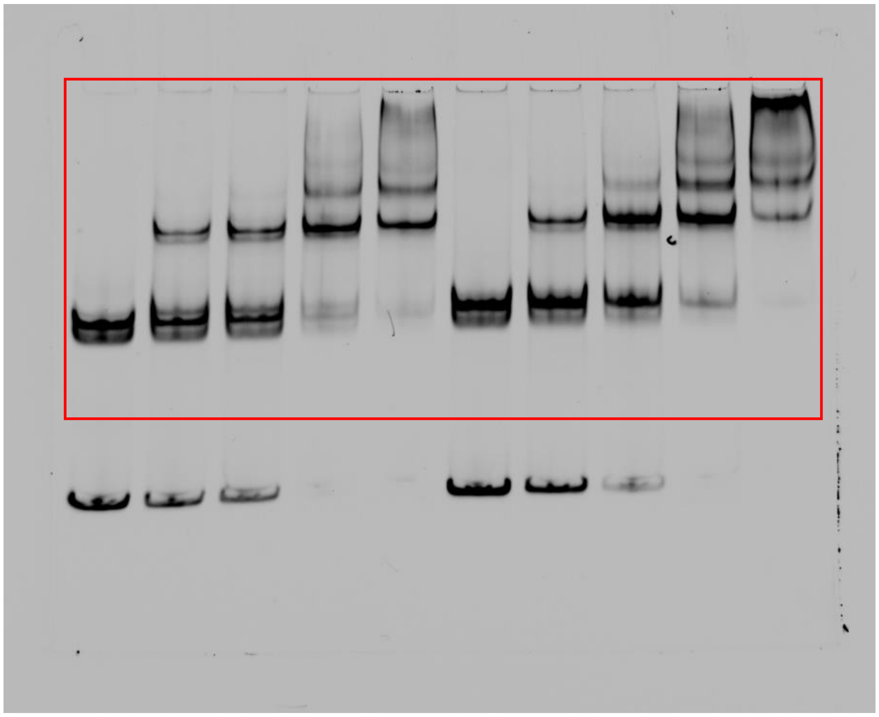

Figure 3e

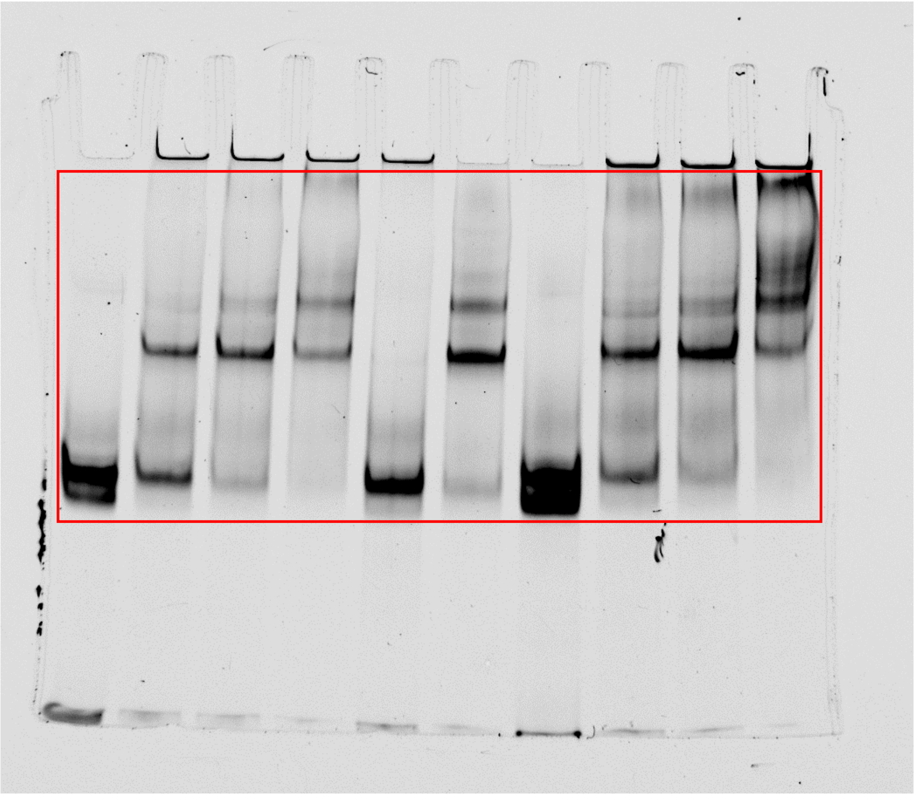

Figure 4a

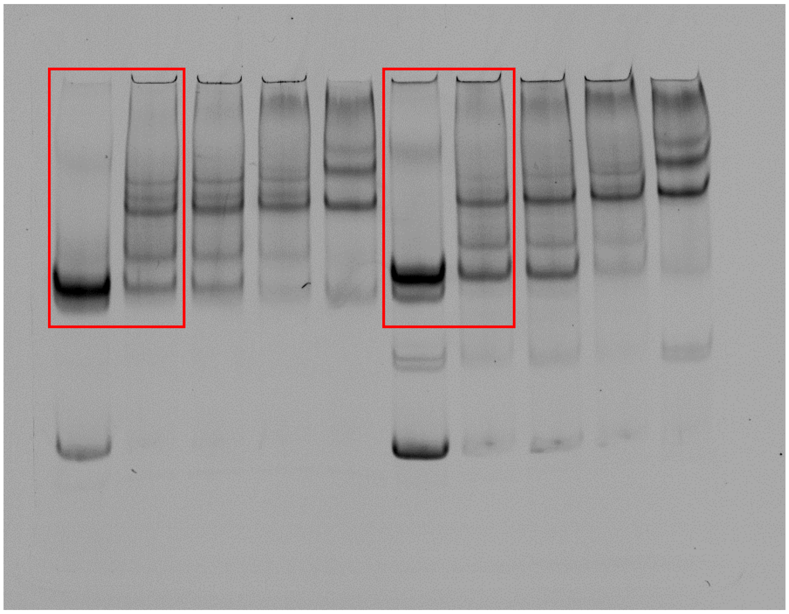

Figure 4b

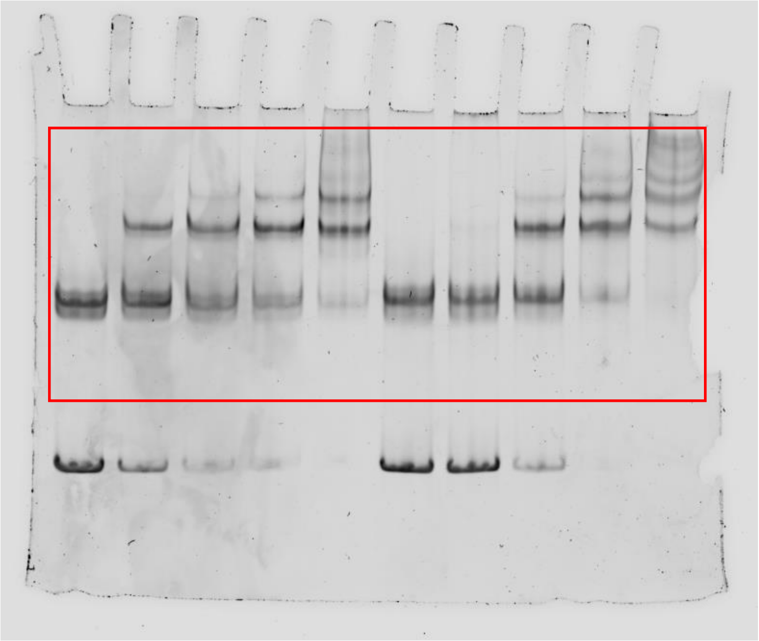

Figure 5c

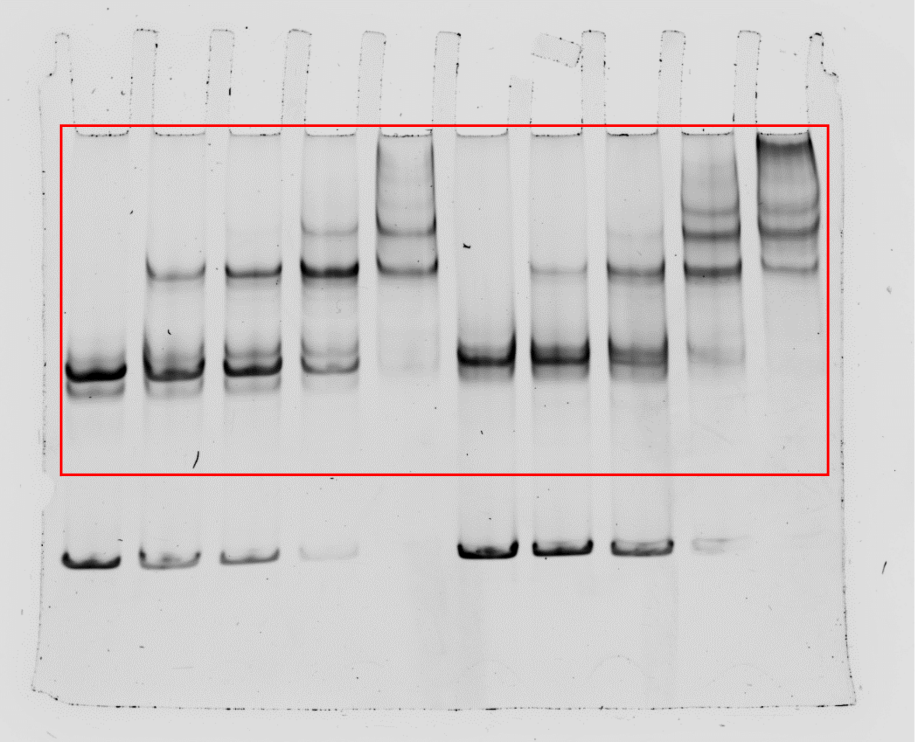

Figure 5d

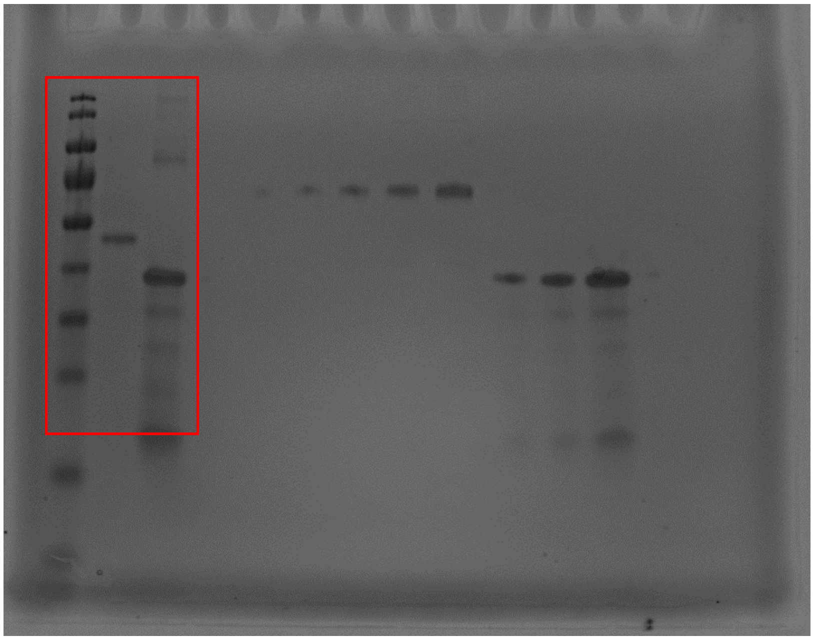

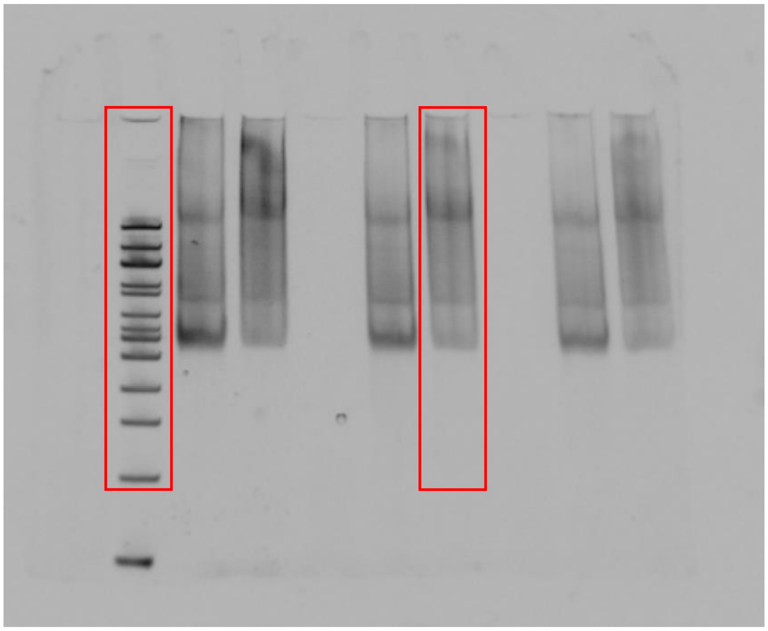

Extended Data 1a

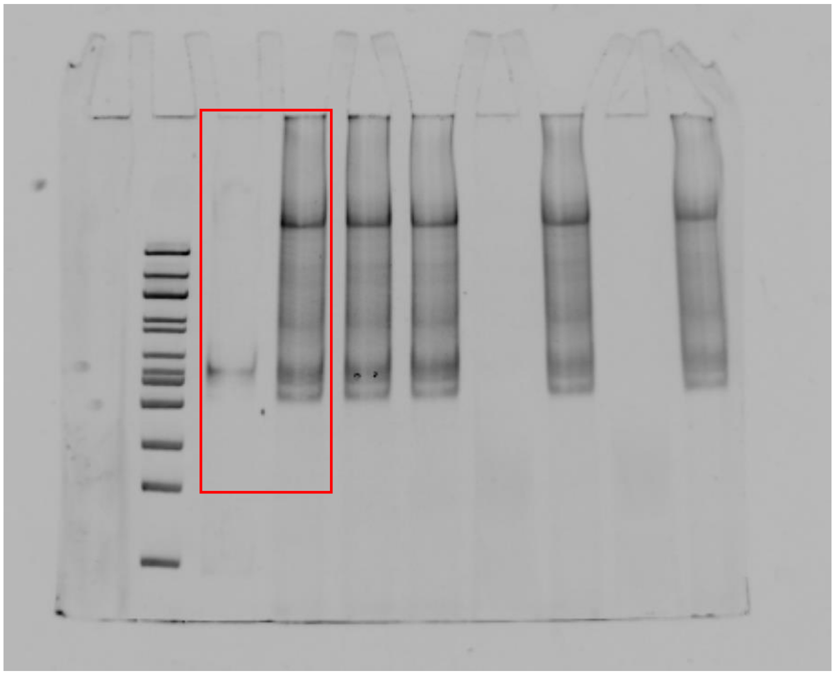

DNA

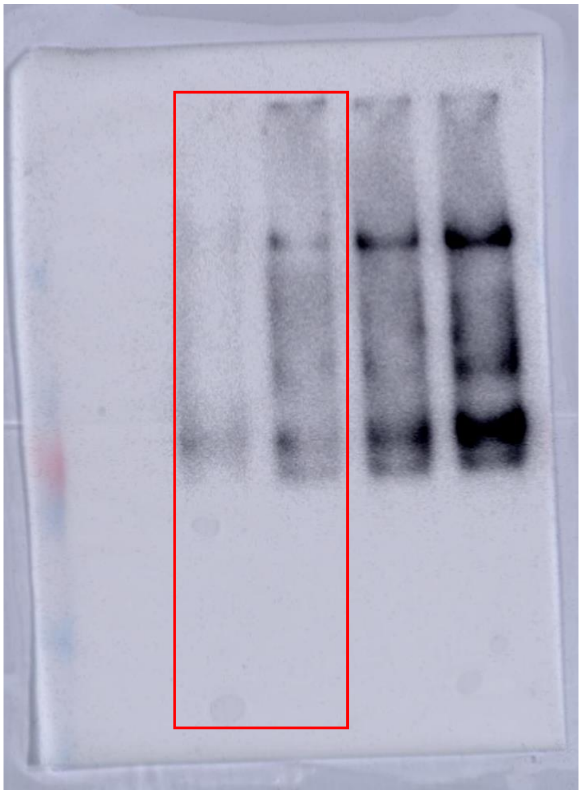

Anti-H3

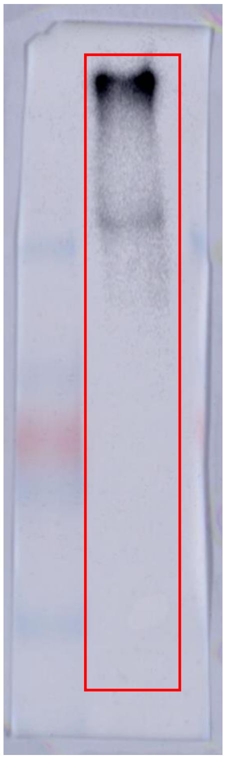

anti-Oct4

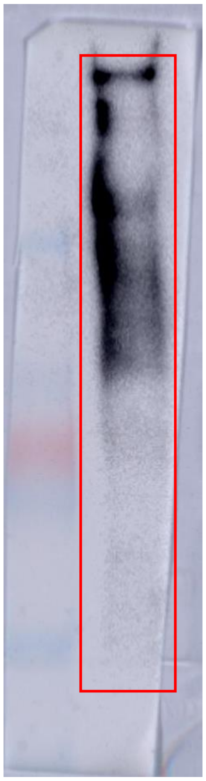

anti-Sox2

Extended Data 1a

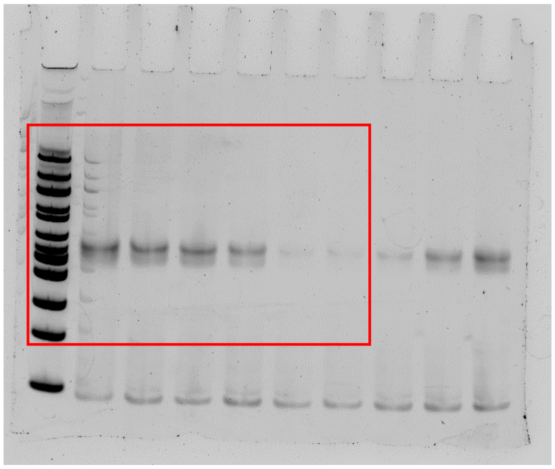

nucleosome

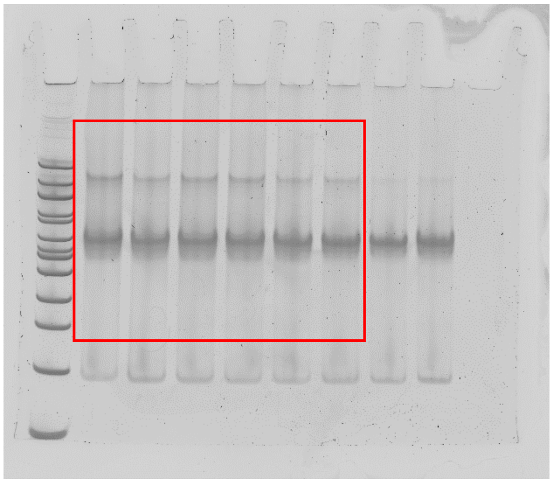

Oct4

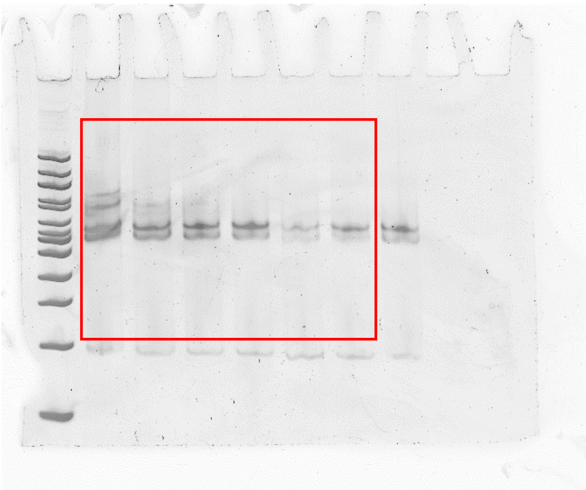

Oct4ΔNtail

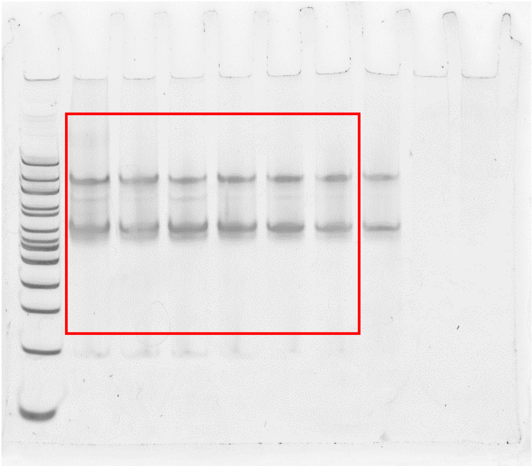

Oct4ΔCtail

Extended Data 5a

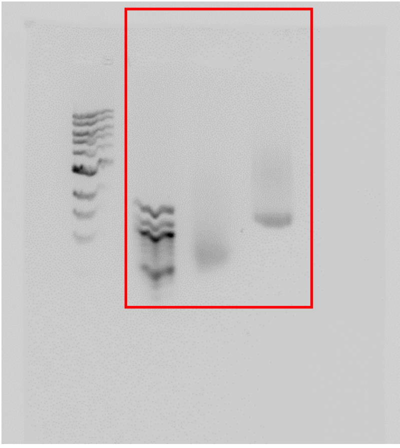

Extended Data 5b

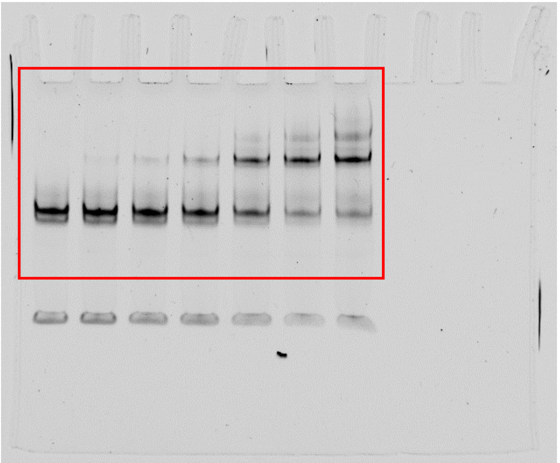

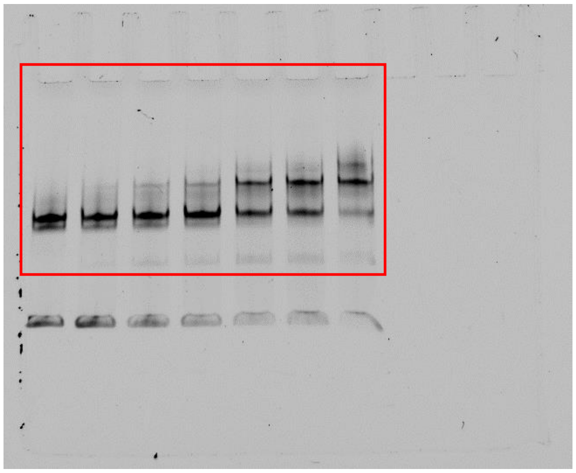

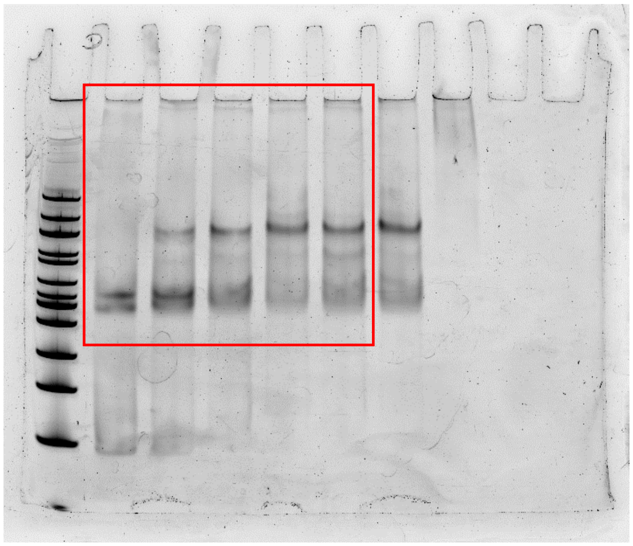

Extended Data 5d

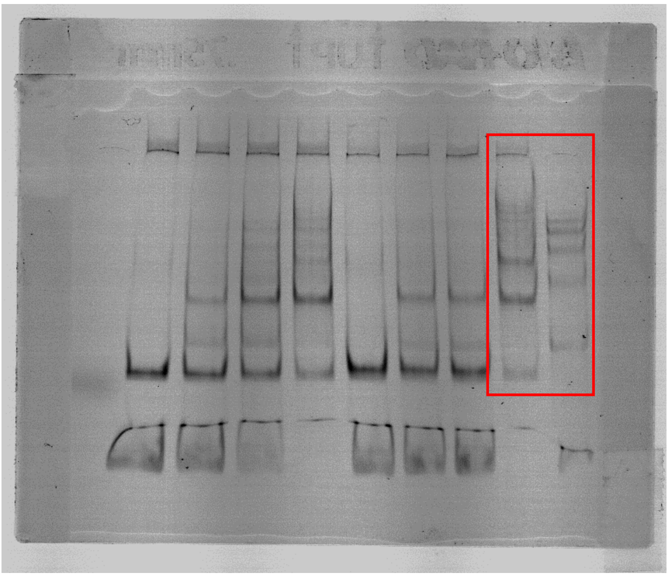

Extended Data 5f

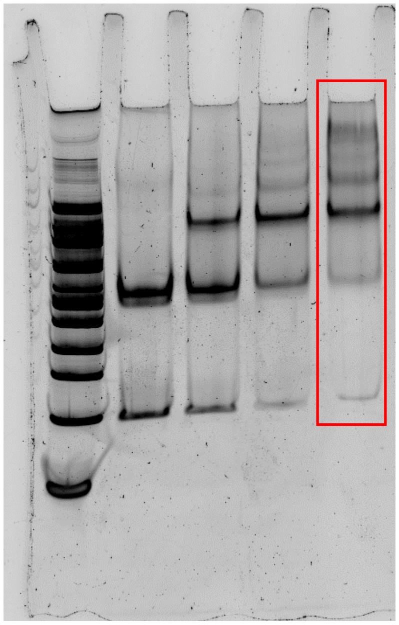

DNA

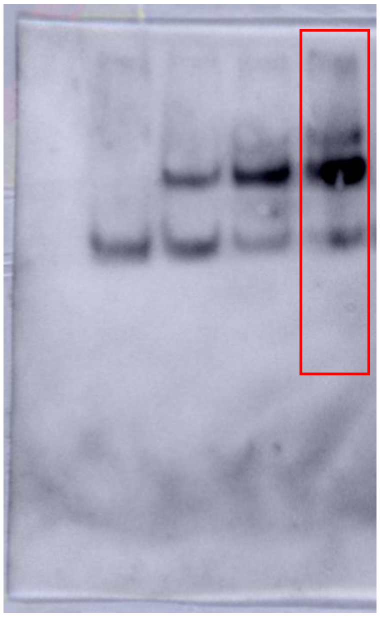

Anti-H3

Extended Data 5g

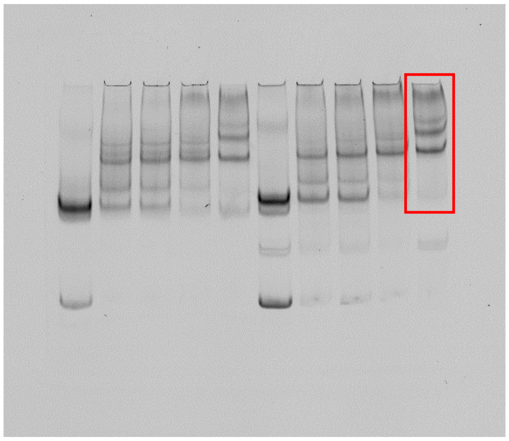

DNA

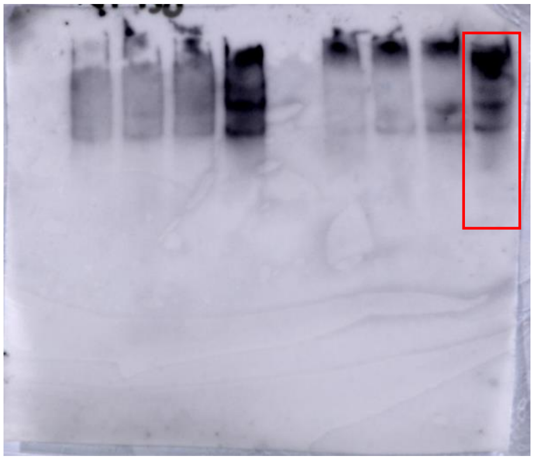

anti-Oct4

Extended Data 5g

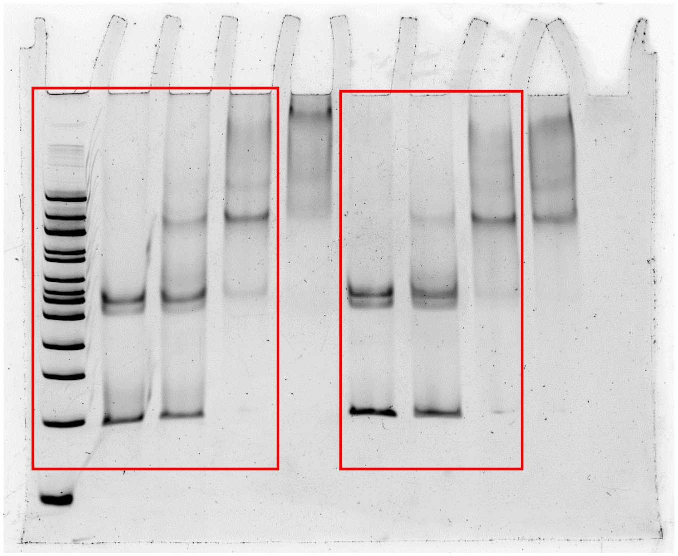

LIN28B LIN28B_2M

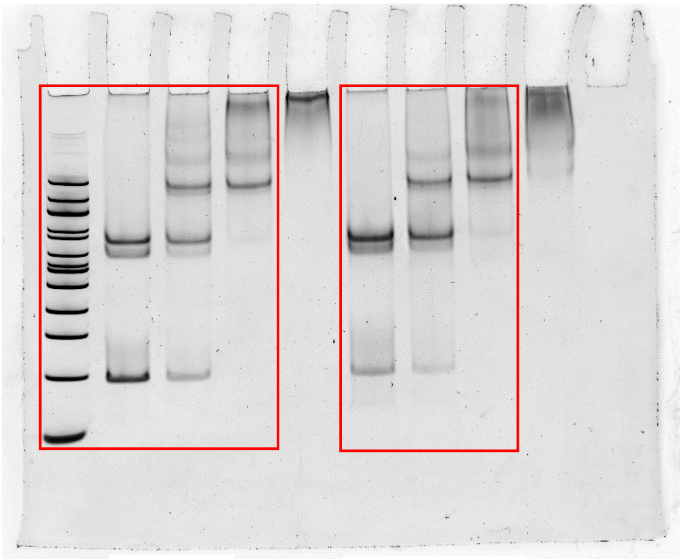

LIN28B LIN28B_3M

Extended Data 5h

Extended Data 5i

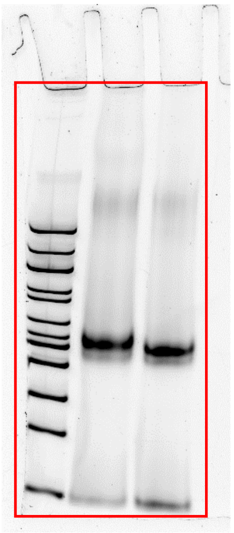

Extended Data 6a

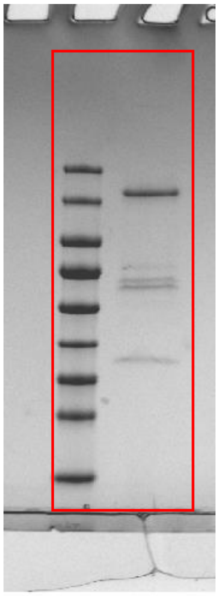

MIDAC

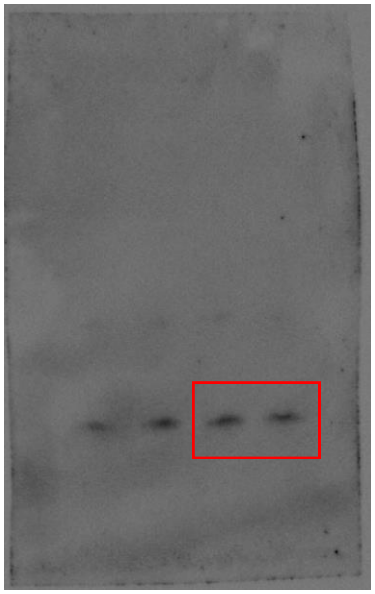

anti-H3

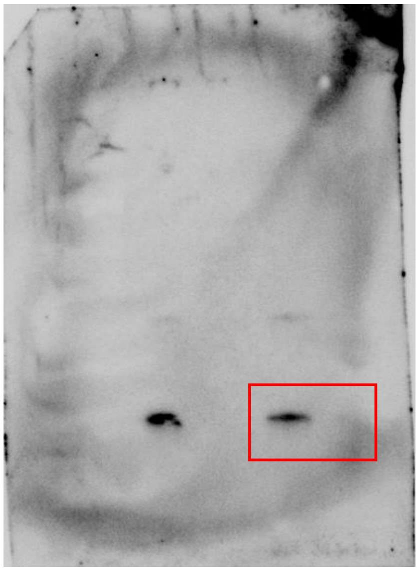

anti-H3K27ac

Extended Data 6b

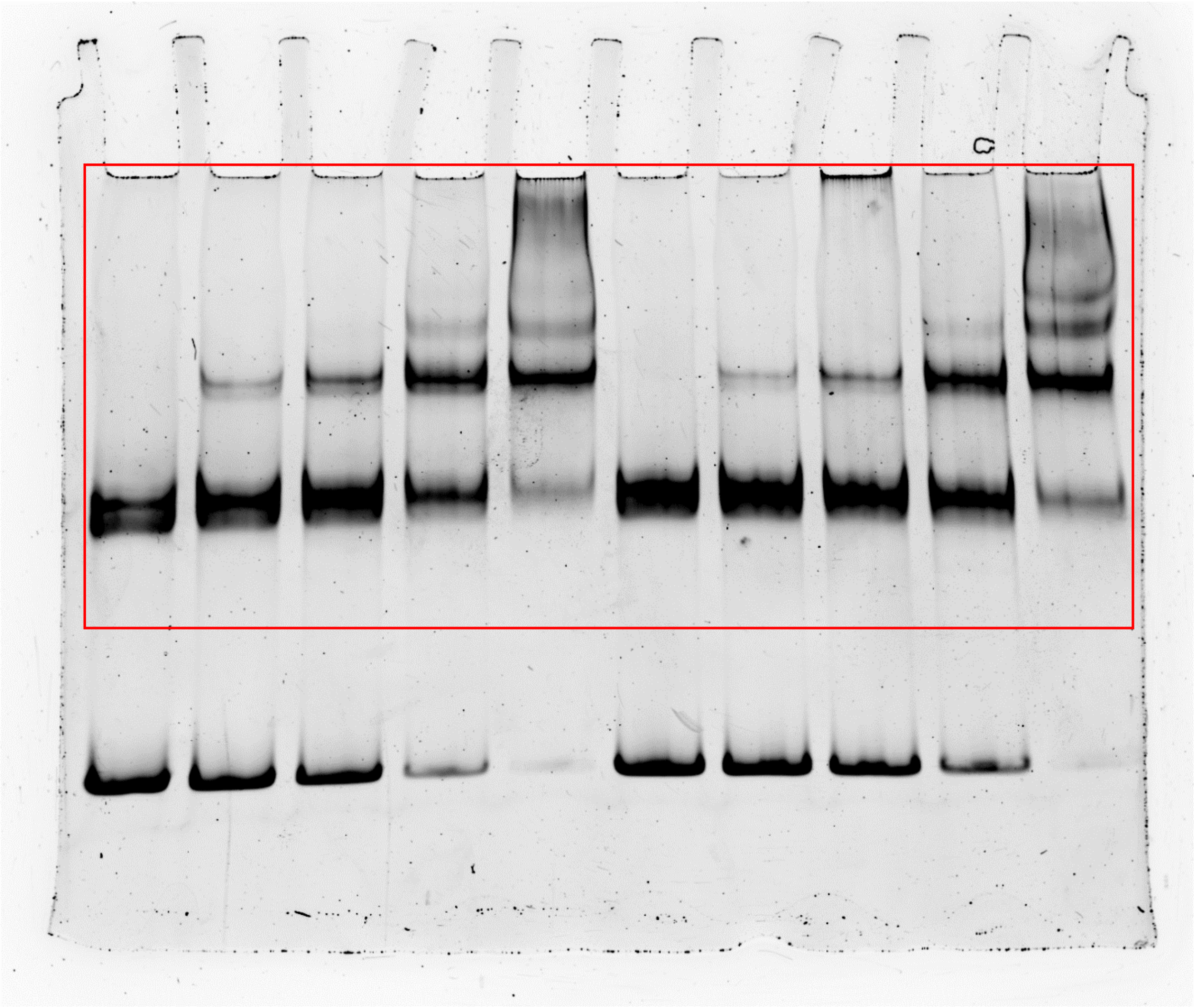

Extended Data 6c

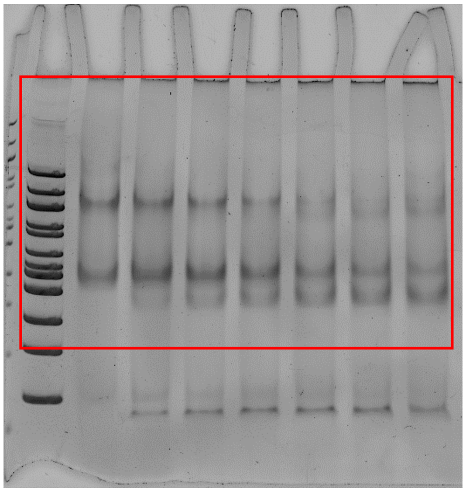

Oct4_Lin28B

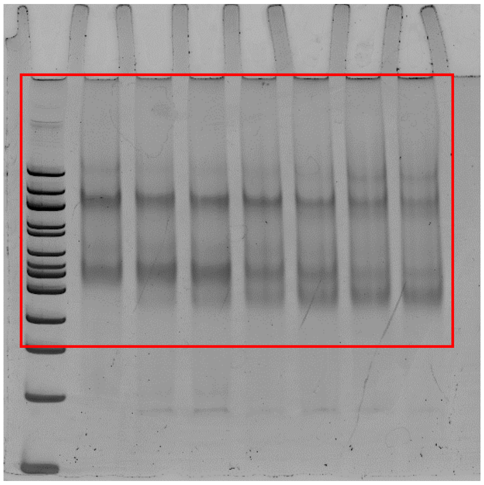

Oct4_Lin28B-H3K27ac

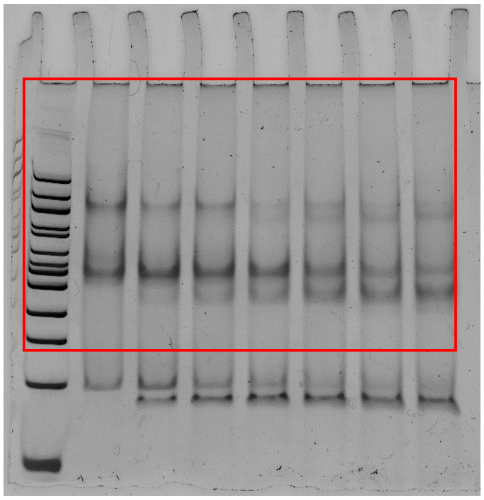

Oct4_Lin28B-H3Kme3

Extended Data 6d

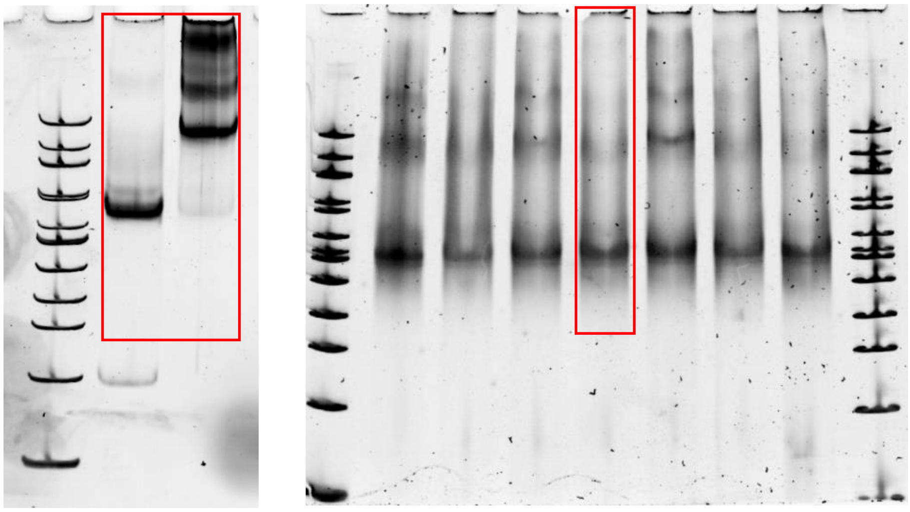

LIN28B

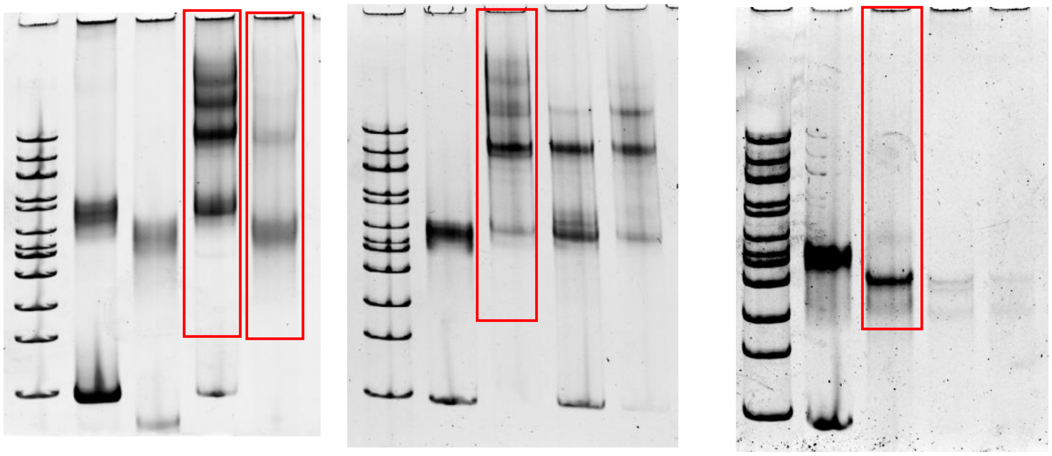

LIN28B H3K27ac

LIN28B H3K27me3

Extended Data 6d

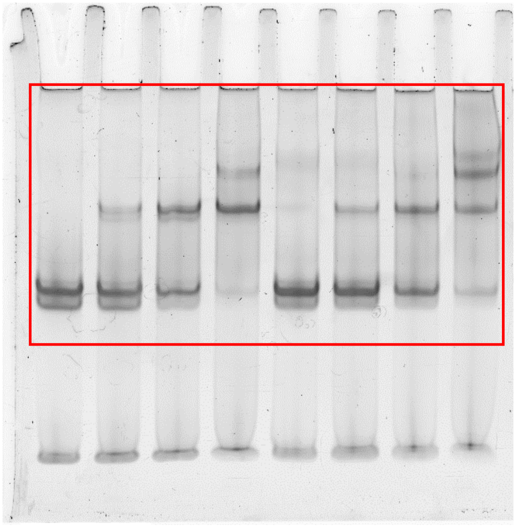

LIN28B LIN28B_ OSO+1

Extended Data 6h

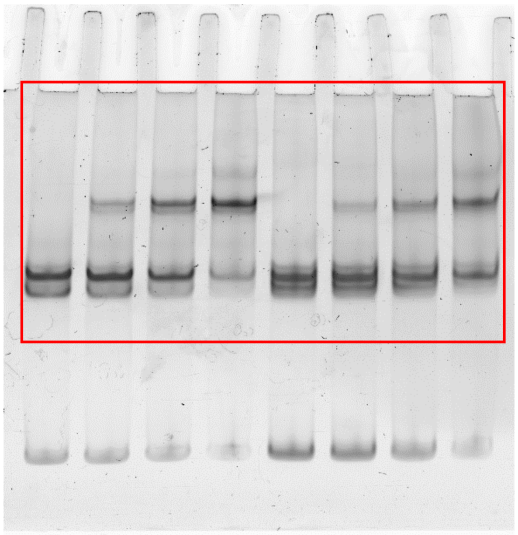

LIN28B LIN28B_ OSO+2

Extended Data 6i

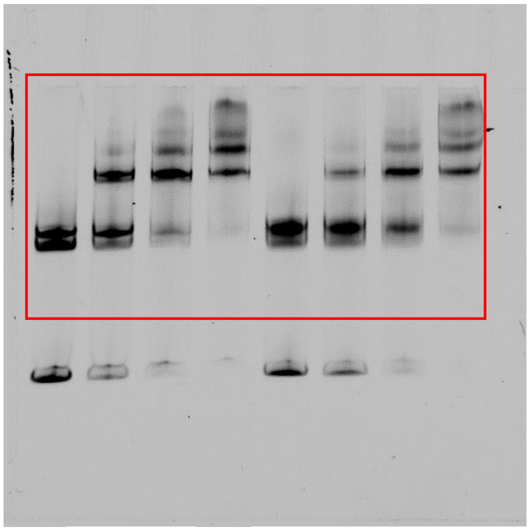

LIN28B LIN28B_ H3K27ac OSO+1

Extended Data 6J

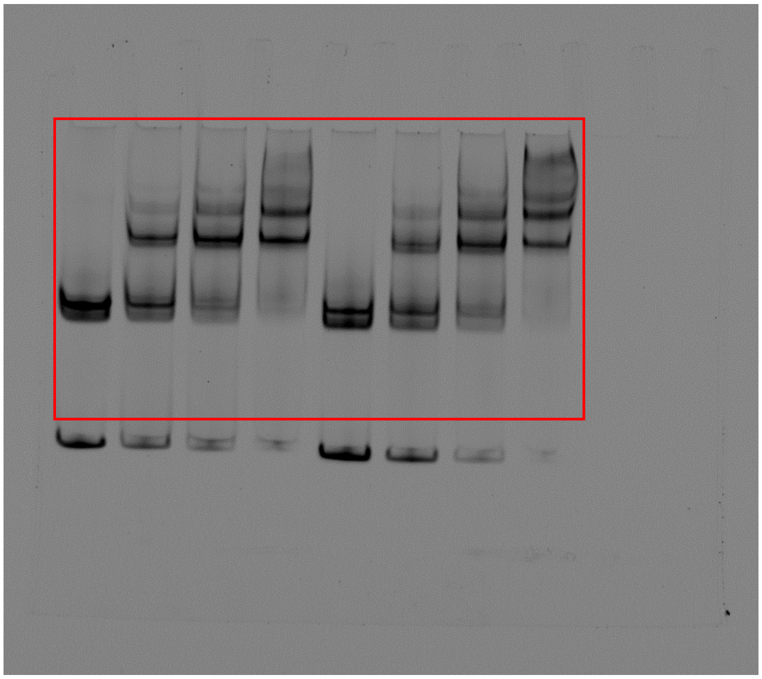

Extended Data 7a

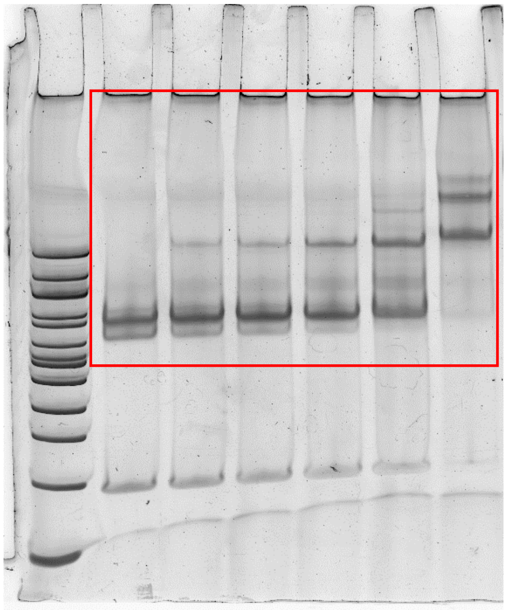

Extended Data 7e

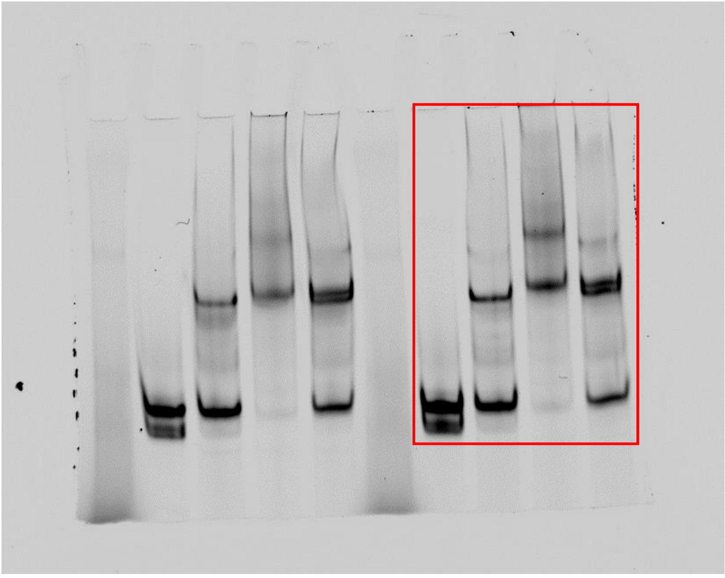

DNA

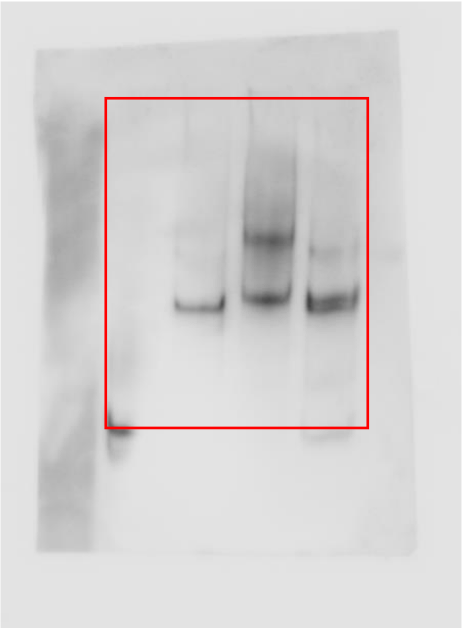

anti-H3

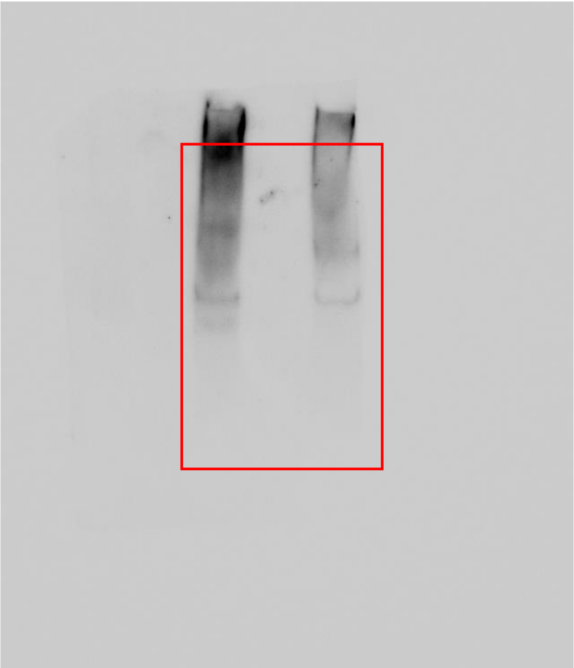

anti-Sox2

Extended Data 7f

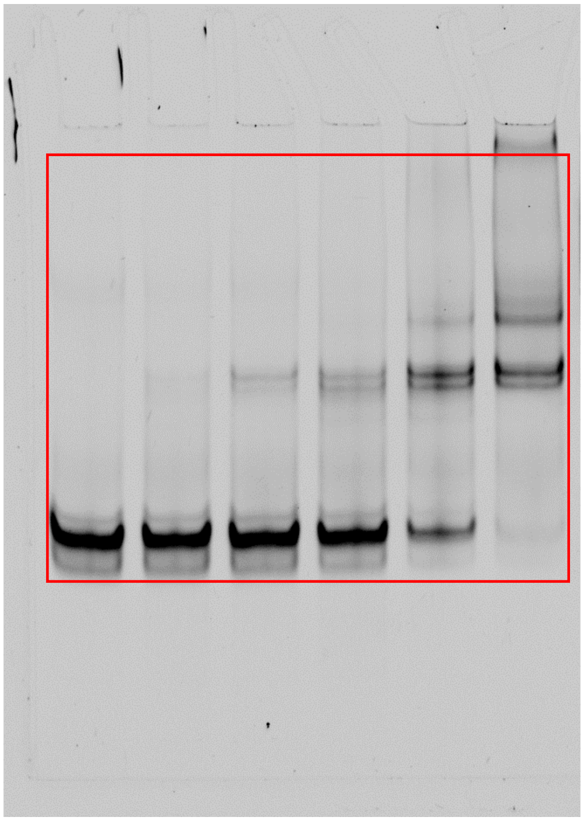

Extended Data 7g

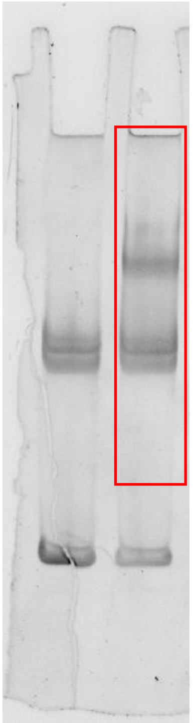

Extended Data 8a

Supplementary Table 1: List of oligonucleotides used in the study

**Oligonucleotides**	**Sequence**
**LIN28 amplification primers**	
LIN28B 1010 Forward primer	GCATAAGTTAAGT GGT ATT AAC ATA TCC TCA GTG GTG
LIN28B 1010 Reverse primer	ATGAAGTATGTGT CTT TAT TCA CAA GCT TGC ACA A
LIN28B_1M Forward primer	GCATAAGTTAAGT GGT GCGTCG ATA TCC TCA GTG GTG
LIN28B_1M Reverse primer	ATGAAGTATGTGT CTT TAT TCA CAA GCT TGC ACA A
LIN28B 1010 Forward primer - Biotinylated	/5Biosg/GATAT CAG TTG GAG CAT AAG TTA AGT GGT ATT AAC ATA
Forward primer for LIN28B genomic array DNA	TATTATGATATTTAATGCATCTGTG
Reverse primer for LIN28B genomic array DNA	GGCCAATTTAAAGAACTCAATAG
nMatn1_F	ACATGCACACATGCTAATATATG
nMatn1_R	TGCATGTATGTGTATGCATATGC
**Primers for Oct4 mutagenesis**	
Oct4_delN_For	CAGGACATCAAAGCTCTG
Oct4_delN_Rev	CATCGAATTCGGATCCCGAC
Oct4_delC_For	CCCATGCATTCAAACTGA
Oct4_delC_Rev	TGATCGCTTGCCCTTCTG
**DNA sequences**	
Lin28B	5': GCAT AAG TTA AGT GGT ATT AAC ATA TCC TCA GTG GTG AGT ATT AAC ATG GAA CTT ACT CCA ACA ATA CAG ATG CTG AAT AAA TGT AGT CTA AGT GAA GAA AGA AGG AAA GGT GGG AGC TGC CAT CAC TCA GAA TTG TCC AGC AGG GAT TGT GCA AGC TTG TGA ATA AAG ACA CAT ACT TCA T;3'
Lin28B locus	TATTATGATATTTAATGCATCTGTGGCTTAACACTTGTGAGAGTTAC CAGCTTGAAAATGATGGTGTTGACTACCTCTTGAATCACATCTATC AACCACTGGCACCTACCACCAAGCTGGCTTCAATTAGTATGTGTT GCTTTTTGGTATTAACAACTAACCGTACTAGAGACCAAAGTGAAC CCTGATTTTTATATGTCTTTAATAATGGTGTTTTATCTAGTGTTTTTA AATTATCCTGTGTAGTATTTAGATTACCTCATTGTCCATTTTGACTC ATGTTGTTTACAAGTGAAAATAAAAACACTTGAACTGTATGTTTTTA AAAGACAAAAAAGGGGTAGATGTTTGGAATGCGTTTCACTCGCAT GCAGTCATCTGGAGGGACTGAAGCACTGTTTGCCTTTCTGTACA CTCTGGGTTTTATATTCTCATTTCATGCCTAATGTCTTATTCTGTCA ATTATGGATATGTTGAGGTTTAAAAAAATTACTTGATTAAAAATAAAA CATATAACGTTGGCATTTATGATGGGTTTTGGAGATTTTTTATGATG

	TGTGTTCTTACTAAGTTAAGTGAAAGGATAAAAGGCCATTTAGGCA<br>GTGTTTTACATCAGTTGGA**GCATAAGTTAAGTGGTATTAACATATC**<br>**CTCAGTGGTGAGTATTAACATGGAACTTACTCCAACAATACAGA**<br>**TGCTGAATAAATGTAGTCTAAGTGAAGGAAGAAGGAAAGGTGG**<br>**GAGCTGCCATCACTCAGAATTGTCCAGCAGGGATTGTGCAAGC**<br>**TTGTGAATAAAGACACATACTTCA**TGTAGTCAGAAGAGTGGTCT<br>GAAGCAAAATATTCAAAGCCTATTGAAGAAATCATTTTAGAATTTTT<br>CTACAAGTTTTTTCTAATAAAGAATTTGAAAAGGGCAAATTTTATCA<br>GTACCAAATATCTAAAATACGAATGTAGATTTATCAAACCATGAATG<br>CAGTATTGATGTACTGTAACAAACGATTTGGTAAAACTATTGAGTT<br>CTTTAAATTGGCC
OBS (+) 1 bp DNA:	GCAT AAG TTA AGT GGT ATT AAC ATA TCC TCA GTG GTG AGT<br>**A** ATT AAC ATG GAA CTT ACT CCA ACAATA CAG ATG CTG<br>AATAA TGT AGT CTA AGT GAA GAA AGA AGG AAA GGT GGG<br>AGC TGC CAT CAC TCAGAA TTG TCC AGC AGG GAT TGT GCA<br>AGC TTG TGA ATA AAG ACA CAT ACT TCA T
OBS (+) 2 bp DNA:	GCAT AAG TTA AGT GGT ATT AAC ATA TCC TCA GTG GTG AGT<br>**AA** ATT AAC ATG GAA CTT ACT CCA ACAATA CAG ATG CTG<br>AATA TGT AGT CTA AGT GAA GAA AGA AGG AAA GGT GGG<br>AGC TGC CAT CAC TCAGAA TTG TCC AGC AGG GAT TGT GCA<br>AGC TTG TGA ATA AAG ACA CAT ACT TCA T
SBS (-) 5 bp DNA	GCAT AAG TTA AGT GGT ATT AAC ATA TCC TCA GTG GTG AGT<br>ATT AAC AT G GAA C TT A ACAATA **ACT CC** CAG ATG CTG AA T<br>AAA T GT AGT CTA AGT GAA GAA AGA AGG AAA GGT GGG AGC<br>TGC CAT CAC TCAGAA TTG TCC AGC AGG GAT TGT GCA AGC<br>TTG TGA ATA AAG ACA CAT ACT TCA T
nMatn1	ACATGCACACATGCTAATATATGCACACAATGCACACAGGTTAATA<br>TATACACATACACACACATGCACACACACGTGCACACATATATGCA<br>CATGCATGCACACACGTATATGCACACACATGCACATGCATGCGC<br>ACATAGTCACACACATGCACACATTAGCATATGCATACACATACAT<br>GCA

Supplementary Table 2: Plasmids used in this study

SJ131	pDeuet-LIN28B
SJ134	pET28-Oct4 N-terminus deletion mutant
SJ135	pET28-Oct4 C-terminus deletion mutant

Supplementary Table 3: Table with quantification of data

Figure 3b

<table>
<tr><td></td><td>normalized to input nucl</td><td>binding to OBS2/3</td></tr>
<tr><td colspan="3">Lin28B</td></tr>
<tr><td>input nucleosome (black)</td><td>1</td><td></td></tr>
<tr><td>nucl. with 1 Oct4 (red)</td><td>0.32</td><td rowspan="2">ratio [2 Oct4/1 Oct4]
0.34</td></tr>
<tr><td>nucl. with 2 Oct4 (blue)</td><td>0.11</td></tr>
<tr><td colspan="3">Lin28B_1M</td></tr>
<tr><td>input nucleosome (black)</td><td>1</td><td rowspan="2">ratio [1 Oct4/nucleosome]
0.07</td></tr>
<tr><td>nucl. with 1 Oct4 (red)</td><td>0.07</td></tr>
</table>

Figure ED 7g

<table>
<tr><td></td><td>normalized to input nucl</td><td>binding to OBS2/3</td></tr>
<tr><td>input nucleosome (black)</td><td>1</td><td rowspan="2">ratio [Sox2/nucleosome]
0.13</td></tr>
<tr><td>Sox2 (grey)</td><td>0.13</td></tr>
<tr><td>1_Oct4 (red)</td><td>0.23</td><td rowspan="2">ratio [1 Oct4_Sox2/1_Oct4]
0.48</td></tr>
<tr><td>1_Oct4_Sox2 (orange)</td><td>0.11</td></tr>
</table>
