# Supplementary material for: Histone modifications regulate pioneer transcription factor cooperativity
Source: Nature. 2023 May 24;619(7969):378–84. doi: 10.1038/s41586-023-06112-6 (PMC10338341; doi:10.1038/s41586-023-06112-6)
Supplement: Supplementary file 2 — Reporting Summary [file 41586_2023_6112_MOESM2_ESM.pdf]

nature portfolio

Corresponding author(s): Mario Halic

Last updated by author(s): 03-19-2023

Reporting Summary

Nature Portfolio wishes to improve the reproducibility of the work that we publish. This form provides structure for consistency and transparency in reporting. For further information on Nature Portfolio policies, see our Editorial Policies and the Editorial Policy Checklist.

Statistics

For all statistical analyses, confirm that the following items are present in the figure legend, table legend, main text, or Methods section.

n/a	Confirmed	
☐	☒	The exact sample size (*n*) for each experimental group/condition, given as a discrete number and unit of measurement
☒	☐	A statement on whether measurements were taken from distinct samples or whether the same sample was measured repeatedly
☐	☒	The statistical test(s) used AND whether they are one- or two-sided<br>*Only common tests should be described solely by name; describe more complex techniques in the Methods section.*
☒	☐	A description of all covariates tested
☒	☐	A description of any assumptions or corrections, such as tests of normality and adjustment for multiple comparisons
☐	☒	A full description of the statistical parameters including central tendency (e.g. means) or other basic estimates (e.g. regression coefficient) AND variation (e.g. standard deviation) or associated estimates of uncertainty (e.g. confidence intervals)
☐	☒	For null hypothesis testing, the test statistic (e.g. *F*, *t*, *r*) with confidence intervals, effect sizes, degrees of freedom and *P* value noted<br>*Give P values as exact values whenever suitable.*
☒	☐	For Bayesian analysis, information on the choice of priors and Markov chain Monte Carlo settings
☒	☐	For hierarchical and complex designs, identification of the appropriate level for tests and full reporting of outcomes
☒	☐	Estimates of effect sizes (e.g. Cohen's *d*, Pearson's *r*), indicating how they were calculated

Our web collection on statistics for biologists contains articles on many of the points above.

Software and code

Policy information about availability of computer code

Data collection	Serial EM for data collection
Data analysis	Relion, CtfFind, Phenix, MotionCor, Coot

For manuscripts utilizing custom algorithms or software that are central to the research but not yet described in published literature, software must be made available to editors and reviewers. We strongly encourage code deposition in a community repository (e.g. GitHub). See the Nature Portfolio guidelines for submitting code & software for further information.

Data

Policy information about availability of data

All manuscripts must include a data availability statement. This statement should provide the following information, where applicable:

- Accession codes, unique identifiers, or web links for publicly available datasets
- A description of any restrictions on data availability
- For clinical datasets or third party data, please ensure that the statement adheres to our policy

EM densities and models have been deposited in the Electron Microscopy Data Bank and PDB under accession codes P DB 8G8G for Oct4 bound to Lin28B nucleosome built using maps EMD-29855 (Oct4 _Nucleosome, all particles), EMD-29850 (Oct4 _Nucleosome, H3 tail subset), E M D-29852 (Oct4 _Nucleosome, H2A tail subset), EMD-29853 (Oct4 _Nucleosome, H4 tail A subset), EMD-29854 (Oct4 _Nucleosome, H4 tail B subset), EMD-29846 (Oct4 _Nucleosome, Oct4 focus) and

PDB 8G8E. For Oct4 bound to n Matnl nucleosome following maps and coordinates were deposited: EMD-29837 and PDB 8G86 (Oct4 _Nucleosome, nucleosome focus); EMD-29841 and PDB 8G87 (Oct4 _Nucleosome, Oct4 focus); EMD-29843 and PDB 8G88 (Oct4_Nucleosome, conformation 1); EMD-29845 and PDB 8G8B (Oct4 _Nucleosome, conformation 2).

Human research participants

Policy information about studies involving human research participants and Sex and Gender in Research.

Reporting on sex and gender	NA
Population characteristics	NA
Recruitment	NA
Ethics oversight	NA

Note that full information on the approval of the study protocol must also be provided in the manuscript.

Field-specific reporting

Please select the one below that is the best fit for your research. If you are not sure, read the appropriate sections before making your selection.

☒ Life sciences ☐ Behavioural & social sciences ☐ Ecological, evolutionary & environmental sciences

For a reference copy of the document with all sections, see nature.com/documents/nr-reporting-summary-flat.pdf

Life sciences study design

All studies must disclose on these points even when the disclosure is negative.

Sample size	This has been described in the corresponding figure legends. No sample size calculation was performed. For the biochemical experiments, we have performed independent experiments, which is sufficient to establish the variation.
Data exclusions	In the EM analysis, only junk particles have been removed using 2D and 3D classifications in RELION. No other data exclusions involved in analysis.
Replication	Biochemical assays have been replicated and the number of replicates has been mentioned in the figure legends. for each experiment. The cryo-EM structure determination generally does not involve repeat of the experiments, specially because of the final structure is the result of ensemble averaging of several thousand particles.
Randomization	Because there was no sub-group analysis involved and the sample size was small, no randomization was done.
Blinding	Blinding was not applicable to this study. Biochemical experiments were visualized using fluorescence and quantification of the bands was done using software (Quantity One, Biorad), requiring no subjective analysis. Likewise, cryoEM data was imaged and processed using standard software packages requiring no subjective judgement.

Reporting for specific materials, systems and methods

We require information from authors about some types of materials, experimental systems and methods used in many studies. Here, indicate whether each material, system or method listed is relevant to your study. If you are not sure if a list item applies to your research, read the appropriate section before selecting a response.

Materials & experimental systems

n/a	Involved in the study
☐	☒ Antibodies
☐	☒ Eukaryotic cell lines
☐	☐ Palaeontology and archaeology
☐	☐ Animals and other organisms
☐	☐ Clinical data
☐	☐ Dual use research of concern

Methods

n/a	Involved in the study
☐	☐ ChIP-seq
☐	☐ Flow cytometry
☐	☐ MRI-based neuroimaging

Antibodies

Antibodies used	anti-Oct4 antibody (Abcam ab109183), HRP-conjugated anti-His antibody (Invitrogen – Thermo Fisher R931-25), anti-H3 antibody (abcam ab1791), anti-Sox2 antibody (Abcam ab 92494), HRP-conjugated anti-rabbit secondary antibody (Biorad 170-6515)
Validation	anti-Oct4 antibody (abcam ab109183):<br>validation for Oct4 band in NCCIT (Human pluripotent embryonic carcinoma epithelial cell) whole cell lysate<br>(https://www.abcam.com/oct4-antibody-epr2054-ab109183.html)<br><br>HRP-conjugated anti-His antibody (Invitrogen – Thermo Fisher, Catalog # R931-25):<br>https://www.thermofisher.com/antibody/product/6x-His-Tag-Antibody-clone-3D5-Monoclonal/R931-25<br><br>anti-H3 antibody (abcam ab1791)<br>Chromatin from Xenopus laevis oocytes, A431 (Human epithelial carcinoma cell line) Whole Cell Lysate, Jurkat (Human T cell lymphoblast-like cell line) Whole Cell Lysate, HEK293 (Human embryonic kidney cell line) Whole Cell Lysate<br>(https://www.abcam.com/histone-h3-antibody-nuclear-marker-and-chip-grade-ab1791.html)<br><br>anti-Sox2 antibody (abcam ab 92494)<br>NCCIT (human pluripotent embryonic carcinoma cell line) whole cell lysate, MCF7 (human breast adenocarcinoma cell line) whole cell lysate, Human glioma lysate<br>(https://www.abcam.com/sox2-antibody-epr3131-ab92494.html)<br><br>HRP-conjugated anti-rabbit secondary antibody (Biorad, 170-6515):<br>https://www.bio-rad.com/en-us/sku/1662408EDU-secondary-antibody-goat-anti-rabbit-antibody-conjugated-horseradish-peroxidase?ID=1662408EDU

Eukaryotic cell lines

Policy information about cell lines and Sex and Gender in Research

Cell line source(s)	Flp-In™ T-REx™ 293 Cell Line (Catalogue # R78007) from Thermo Fischer Scientific
Authentication	Authenticated by STR profiling at St Jude Children's Research Hospital
Mycoplasma contamination	Tested negative for Mycoplasma contamination.
Commonly misidentified lines (See ICLAC register)	None

Palaeontology and Archaeology

Specimen provenance	NA
Specimen deposition	NA
Dating methods	NA

☐ Tick this box to confirm that the raw and calibrated dates are available in the paper or in Supplementary Information.

Ethics oversight	NA

Note that full information on the approval of the study protocol must also be provided in the manuscript.

Animals and other research organisms

Policy information about studies involving animals; ARRIVE guidelines recommended for reporting animal research, and Sex and Gender in Research

Laboratory animals	NA
Wild animals	NA
Reporting on sex	NA
Field-collected samples	NA
Ethics oversight	NA

Note that full information on the approval of the study protocol must also be provided in the manuscript.

Clinical data

Policy information about clinical studies

All manuscripts should comply with the ICMJE guidelines for publication of clinical research and a completed CONSORT checklist must be included with all submissions.

Clinical trial registration	NA
Study protocol	NA
Data collection	NA
Outcomes	NA

Dual use research of concern

Policy information about dual use research of concern

Hazards

Could the accidental, deliberate or reckless misuse of agents or technologies generated in the work, or the application of information presented in the manuscript, pose a threat to:

No	Yes	
☒	☐	Public health
☒	☐	National security
☒	☐	Crops and/or livestock
☒	☐	Ecosystems
☒	☐	Any other significant area

Experiments of concern

Does the work involve any of these experiments of concern:

No	Yes	
☒	☐	Demonstrate how to render a vaccine ineffective
☒	☐	Confer resistance to therapeutically useful antibiotics or antiviral agents
☒	☐	Enhance the virulence of a pathogen or render a nonpathogen virulent
☒	☐	Increase transmissibility of a pathogen
☒	☐	Alter the host range of a pathogen
☒	☐	Enable evasion of diagnostic/detection modalities
☒	☐	Enable the weaponization of a biological agent or toxin
☒	☐	Any other potentially harmful combination of experiments and agents

ChIP-seq

Data deposition

☐ Confirm that both raw and final processed data have been deposited in a public database such as GEO.

☐ Confirm that you have deposited or provided access to graph files (e.g. BED files) for the called peaks.

Data access links<br>*May remain private before publication.*	NA
Files in database submission	NA
Genome browser session<br>(e.g. UCSC)	NA

Methodology

Replicates	NA

Sequencing depth	NA
Antibodies	NA
Peak calling parameters	NA
Data quality	NA
Software	NA

Flow Cytometry

Plots

Confirm that:

☐ The axis labels state the marker and fluorochrome used (e.g. CD4-FITC).

☐ The axis scales are clearly visible. Include numbers along axes only for bottom left plot of group (a 'group' is an analysis of identical markers).

☐ All plots are contour plots with outliers or pseudocolor plots.

☐ A numerical value for number of cells or percentage (with statistics) is provided.

Methodology

Sample preparation	NA
Instrument	NA
Software	NA
Cell population abundance	NA
Gating strategy	NA

☐ Tick this box to confirm that a figure exemplifying the gating strategy is provided in the Supplementary Information.

Magnetic resonance imaging

Experimental design

Design type	NA
Design specifications	NA
Behavioral performance measures	NA

Acquisition

Imaging type(s)	NA
Field strength	NA
Sequence & imaging parameters	NA
Area of acquisition	NA

Diffusion MRI ☐ Used ☒ Not used

Preprocessing

Preprocessing software	NA
Normalization	NA
Normalization template	NA
Noise and artifact removal	NA

Volume censoring	NA

Statistical modeling & inference

Model type and settings	NA
Effect(s) tested	NA

Specify type of analysis: ☐ Whole brain ☐ ROI-based ☐ Both

Statistic type for inference (See Eklund et al. 2016)	NA
Correction	NA

Models & analysis

n/a	Involved in the study
☒	☐ Functional and/or effective connectivity
☒	☐ Graph analysis
☒	☐ Multivariate modeling or predictive analysis
